# Soluble triggering receptor expressed on myeloid cells-1 as a predictive biomarker in sepsis: insights into cut-offs, mortality risk, and treatment guidance—secondary analysis of the Next GeneSiS-Trial

**DOI:** 10.1186/s13054-026-06025-6

**Published:** 2026-04-21

**Authors:** Vivienne Theobald, Jonas Gregorius, Marc M. Berger, Sebastian O. Decker, Manuel Feißt, Frank Herbstreit, Christian Nusshag, Annabell Skarabis, Karsten D. Schmidt, Felix C. F. Schmitt, Thomas Schmoch, Maximilian Dietrich, Thorsten Brenner, Markus A. Weigand, the German Society of Anaesthesiology and Intensive Care (GSAIC) Trials Group, Yevhen Vainshtein, Silke Grumaz, Mehdi Manoochehri, Andrea Seidel-Glatzer, Mathias W. Pletz, Hendrik Bracht, Kristina Fuest, Manfred Blobner, Friedhelm Bach, Onnen Moerer, Timo Brandenburger, Thomas Dimski, Klaudiusz Suchodolski, Ulrike Jäkel, Jana Zischkau, Helene Häberle, Peter Rosenberger, Tobias Schürholz, Simone Lindau, Stefan J. Schaller, Christian Putensen, Fabian Dusse, Sirak Petros, Max Gaasch, Kai Sohn, Karolina Glanz, Eberhard Barth, Martin S. Winkler, Hans-Jörg Gillmann

**Affiliations:** 1 Department of Anesthesiology, Medical Faculty Heidelberg, Heidelberg University, Im Neuenheimer Feld 420, 69120 Heidelberg, Germany; 2 Department of Anesthesiology and Intensive Care Medicine, University Hospital Essen, University Duisburg-Essen, Essen, Germany; 3 Institute of Medical Biometry, University of Heidelberg, Im Neuenheimer Feld 130.3, 69120 Heidelberg, Germany; 4 Department of Nephrology, Medical Faculty Heidelberg, Heidelberg University, Heidelberg, Germany; 5 Department of Anesthesiology and Intensive Care Medicine, University Hospital Marburg, Philipps University Marburg, 35033 Baldingerstrasse, Marburg, Germany; 6 Department of Anesthesiology and Intensive Care Medicine, Hôpitaux Robert Schuman – Hôpital Kirchberg, Luxembourg City, Luxembourg

**Keywords:** Sepsis, Triggering receptor expressed on myeloid cells-1, Disease severity, Microbial pathogens, Infection site, Cut-off, Treatment guidance, Therapeutic potential, Personalized medicine, Individualized medicine

## Abstract

**Background:**

Triggering receptor expressed on myeloid cells-1 (TREM-1) is one of the more recently described biomarkers for sepsis and currently of great interest, as an inhibitor called nangibotide (Inotrem, Paris, France) for TREM-1 has made it into a phase 3 study for the treatment of sepsis. TREM-1, as a pattern recognition receptor (PRRs), plays a crucial role in the immune defense of the host. Several studies revealed promising results for soluble TREM-1 (sTREM-1) as a biomarker for outcome prediction. However, its usefulness in different subgroups of sepsis and the cut-off threshold at which patients are exposed to a higher risk of mortality have not yet been sufficiently investigated.

**Methods:**

This study is a secondary analysis of data and sTREM-1 measurements from plasma samples obtained in the prospective, observational, non-interventional, multicentric Next GeneSiS-Trial (DRKS00011911). We aimed to characterize the role of sTREM-1 in septic and septic shock patients in a large cohort with regard to different pre-existing factors, infectious aspects and outcome parameters. Furthermore, we investigated the influence of sTREM-1 cut-offs previously described from Francois et al. on different outcome parameters. Therefore, data from 500 patients were analyzed.

**Results:**

Median sTREM-1 values were statistically significant higher in patients with septic shock than in septic patients (406 (IQR;270–650) pg/mL vs. 293 (IQR;189–489) pg/mL; *p* < 0.001). Non-survivors, patients with the need of renal replacement therapy, mechanical ventilation or a positive Sepsis-Induced-Coagulopathy (SIC) score presented with statistically significant higher sTREM-1 values (*p* < 0.001). sTREM-1 levels were significantly higher in patients in the highest quartile for fluid balance and Sequential Organ Failure Assessment score, and significantly lower in those in the highest quartile for Horowitz Index and platelet count (PSSC). sTREM-1 levels showed slight but statistically significant differences based on the site of infection or blood culture results on day one; however, these differences were no longer detectable by day three. The best cut-off of sTREM-1 values to differentiate between survival and non-survival on day 28 was 408 (CI;297–505) pg/ml in our study.

**Conclusion:**

Elevated sTREM-1 concentrations particularly occur in patients with a high degree of organ damage, who are at high risk for adverse outcomes. In addition, the primary site of infection may be of relevance for the sTREM-1 increase. These characteristics support its suitability as a biomarker across different causes of sepsis and highlights its potential as a therapeutic target. A cut-off value exceeding 400 pg/mL has shown robust diagnostic performance across multiple studies. Our results support this observation, indicating that this threshold may be useful for future clinical and research applications.

**Supplementary Information:**

The online version contains supplementary material available at 10.1186/s13054-026-06025-6.

## Background

Sepsis and septic shock are life-threatening conditions that affect 50 million people annually and are responsible for an estimated 11 million deaths each year throughout the world [1]. Therefore, health care systems face the challenge of high personal and financial costs [2] and the development of new diagnostic and therapeutic strategies to improve morbidity and mortality in septic patients remains a crucial task. In the last decades several biomarkers for diagnosing sepsis, differentiation between infection, sepsis, septic shock and sterile systemic inflammatory response syndrome (SIRS), as well as outcome prediction have been described [3] and new biomarkers are emerging nearly on a daily base. However, in clinical routine, C-reactive protein (CRP) and procalcitonin (PCT) are still primarily used for diagnosing infection [4] and almost none of the new ones have been passed into clinical standard care [5]. For outcome prediction, several scores are used in daily routine. Among the most well-known are the Sequential Organ Failure Assessment (SOFA) Score, which can be used for predicting the prognosis of sepsis patients [6] as well as the Acute Physiology And Chronic Health Evaluation-II (APACHE II) score and the Simplified Acute Physiology Score (SAPS) II, which can be used for prediction of mortality on intensive care unit (ICU) and are used for a wide range of diseases [7, 8].

Triggering receptor expressed on myeloid cells-1 (TREM-1) is one of the more recently described biomarkers for sepsis and currently of great interest, as an inhibitor called nangibotide (Inotrem, Paris, France) for TREM-1 has made it into the clinical study phase and is currently being tested in a phase 3 study for the treatment of sepsis [9, 10]. TREM-1 belongs to the family of pattern recognition receptors (PRRs) [11–14] and recognizes pathogen damage-associated molecular patterns (PAMPs/DAMPs). Therefore, TREM-1 plays a crucial role in the immune defense of the host and its activation leads to multiple downstream signal transduction enhancing proinflammatory cytokine secretion [15–17]. Consistently, TREM-1 was first found to be expressed on the surface of monocytes and neutrophils [11], but it is also expressed on non-immune cells like hepatic endothelial cells and gastric epithelial cells.

TREM-1 has become popular in recent years as a candidate for targeted sepsis therapy. The aforementioned nangibotide acts as a ligand-trapping molecule, also called a decoy receptor, that modulates the TREM-1-mediated amplification of inflammatory pathways and the inflammatory response [18]. However, the selection of appropriate patients who could benefit from targeted therapy plays a key role here, and sTREM-1 could be used to stratify patients for this treatment.

The aim of this study was to characterize the role of sTREM-1 in septic and septic shock patients in a large collective with regard to different pre-existing factors, infectious aspects, organ-related outcomes and survival. Our hypothesis is that elevated sTREM-1 levels are predominantly associated with the degree of organ damage, while pre-existing conditions, sex, and immune status play only a secondary role.

## Methods

### Study cohort and definition of endpoints

This was a secondary analysis through evaluating the data from the prospective, observational, non-interventional, multicentric Next GeneSiS-Trial (DRKS00011911) [19] concerning sTREM-1 plasma concentrations in septic and septic shock patients. All patients were enrolled upon admission to the intensive care unit.

The local responsible ethic committee has approved the conduction of this study (Trial Code No. S-084/2017).

Our study is of explorative nature. Therefore, all objectives are considered equivalent. Objectives included the difference of sTREM-1 plasma concentrations at baseline (inclusion) and after 3 days in survivors and non-survivors, in patients with sepsis and those with septic shock, as well as an evaluation of additional outcome parameters, pre-existing conditions and infectious aspects. Furthermore, the analysis included stratification of patients into quartiles based on their SOFA score, fluid balance, Horowitz index, and platelet count. The rationale for this approach was to determine whether specific organ damage is associated with elevated sTREM-1 levels or whether sTREM-1 serves as a general marker of disease severity and organ dysfunction. Additionally, we examined pre-specified cut-off values for high sTREM-1 plasma concentrations in relation to outcomes, disease severity parameters, pre-existing conditions, and 28-day survival.

### Inclusion criteria and outcome definitions

Patients with suspected or proven sepsis/septic shock according to the Sepsis-3 definition with an onset < 24 h before enrolment were eligible for study inclusion. Exclusion criteria included age < 18 years, refusal of consent, probably discharge from ICU within the first 72 h hours following inclusion, palliative treatment or signs for imminent and inevitable death and patients which had previously been included and are readmitted on ICU [19].

Pre-existing conditions of interest included sex, immunosuppression and comorbidities. Immunosuppression was defined as previously described by De Pauw et al. [20]. Concerning infectious aspects, this study focused primarily on the infection onset (nosocomial and outpatient), the infection site and the group of infection causing microorganisms. Outcome parameters included 28-day mortality, the length of intermediate care unit (IMC)/ICU stay, the need and duration of renal replacement therapy (RRT), the need and duration of mechanical ventilation, the length of anti-infective therapy. Furthermore, the correlation of sTREM-1 plasma concentrations with SOFA score, fluid balance, respiratory function (Horowitz index) and coagulation (SIC score and platelet count (PLC) SIC subscore PSSC) was investigated. The SIC score was calculated as suggested by Iba et al. [21]. It was considered positive if two criteria were met simultaneously: (I) a total SIC score ≥ 4 and (II) the sum of the platelet count (PLC) SIC subscore (PSSC) and the international normalized ratio (INR) SIC subscore (ISSC) was ≥ 3 [21, 22]. The SIC score uses a truncated Sequential Organ Failure Assessment (SOFA) score that only considers the sum of the respiratory, cardiocirculatory, hepatic, and renal subscores [21, 22].

Based on previously described cut-offs by Francois et al. [9] (400 pg/mL as “high sTREM-1” plasma concentration), we investigated the influence of sTREM-1 cut-offs on the above-mentioned parameters.

Infections were classified into pulmonary, abdominal, urogenital, and other. Other encompassed bone/soft tissue infections, joint infections, surgical site infections, or combinations of the aforementioned infection sites. Blood cultures (BC) were classified as positive if bacterial or fungal growth was detected within the first 3 days after inclusion. In order to obtain sufficient group sizes, certain types of bacteria were grouped together. The following groups were analyzed separately: Cocci for gram-positive bacterial growth, *Enterobacteriae* for gram-negative bacterial growth, non-fermenters and fungi. This could only be performed for blood cultures taken at inclusion. On day 3, the number of blood cultures positive for fungal or non-fermenter growth was too small for analysis.

### Measurement of sTREM-1

sTREM-1 plasma concentrations were measured at inclusion (Visit 1- V1; timepoint of sepsis diagnosis) and at day 3 after inclusion (Visit 3 - V3). A commercially available enzyme-linked immunosorbent assay (ELISA) was used to measure sTREM-1 plasma concentrations (Quantikine^®^ ELISA, Human TREM-1 Immunoassay, R&D Systems, Inc., USA). The samples were prepared according to the manufacturer’s instructions and diluted for optimal analysis using the provided assay diluent. Measurements were conducted using a Multimode Reader TriStar² LB942 (Berthold Technologies, Bad Wildbad, Germany).

### Statistical methods

Since this is an exploratory secondary analysis, all p-values have to be interpreted in descriptive sense and are not adjusted for multiplicity. No missing values were imputed.

Patients’ characteristics and outcomes were described by median and interquartile range (IQR) for continuous variables, as well as absolute and relative frequencies for categorical variables. Subgroup comparisons were performed using t-tests or chi-squared tests as appropriate. Biomarker values were compared by non-parametric Mann-Whitney-U tests. Correlations were assessed by Pearson’s correlation coefficient. With regard to the interpretation of the correlation coefficients, we have assumed that a correlation coefficient of ≥ 0.3 to < 0.5 indicates a weak, ≥ 0.5 to < 0.8 a moderate, and ≥ 0.8 a strong correlation. Survival was estimated by Kaplan-Meier’s estimate and compared between subgroups by Log-Rank-Tests. Receiver operating characteristics (ROCs) curves were generated to analyze biomarker performances. Best cut-offs were derived from the Youden-Index Method (highest sum of specificity and sensitivity).

##  Results

sTREM-1 plasma concentrations were measured in plasma samples from 500 patients with sepsis or septic shock. sTREM-1 plasma concentrations were in median 366 (241–614) pg/mL at V1 and 302 (201–495) pg/mL at V3 in the overall cohort. Patients were allocated in two different groups: sepsis and septic shock according to the prior defined Sepsis-3 definition **(**Table 1**).** A total of 141 patients were assigned to the sepsis cohort and 359 patients to the septic shock cohort. In patients with septic shock, median sTREM-1 plasma concentration was 406 (270–650) pg/mL and in septic patients 293 (189–489) pg/mL. This difference was statistically significant with *p* < 0.001. Age was similar between both groups. Sex distribution, the Charlson Comorbidity index (CCI) and the immune status between the groups were also comparable. The distribution of infection site was similar. Regarding disease severity and inflammation status, patients with septic shock showed significantly higher SOFA Scores at V1, lactate levels, the leucocyte count and PCT levels. The mortality rate, the need for mechanical ventilation and RRT were significantly higher in the septic shock group, whereas there was no statistical difference concerning the length of ICU stay, the length of mechanical ventilation, the length of anti-infective therapy or the length of RRT (Table 1).

**Table 1:** Baseline characteristics and outcomes

Variable	All patients (*n* = 500) Median (IQR)	*n*	Sepsis (*n* = 141) Median, IQR	*n*	Septic shock (*n* = 359) Median, IQR	*n*	*p* - value
Demographics							
Age, years	68 (58–78)		68 (57–78)		68 (58–78)		0.959
Male, n (%)	337 (67)		93 (66)		244 (68)		0.666
BMI, kg/m^2^	27 (24–31)	491	26 (24–31)	138	27 (24–31)	353	0.932
Comorbidities							
Charlson Comorbidity Index, points	2 (1–4)		2 (1–4)		2 (1–4)		0.159
Immune status							
Immunosuppressed, n (%)	45 (9)		8 (6)		37 (10)		0.103
Disease Severity							
SOFA score, points	9 (7–12)	493	6.5 (5–9)	136	10 (8–12)	357	**< 0.001**
Lactate, mg/dl	18 (12–29)	498	12 (9–16)	140	22 (14–39)	358	**< 0.001**
Infection site; n (%)							**0.029**
Pulmonal	118 (24)		38 (27)		80 (22)		
Gastrointestinal	153 (31)		30 (21)		123 (34)		
Urogenital	66 (13)		18 (13)		48 (13)		
Other	163 (33)		55 (39)		108 (30)		
Origin of infection; n (%)							
Nosocomial	241 (48)		77 (55)		164 (46)		0.074
Infectious source control; n (%)							**< 0.001**
Surgical	239 (48)		42 (30)		197 (55)		
Interventional	50 (10)		23 (16)		27 (8)		
Removal of central venous catheter	26 (5)		13 (9)		13 (4)		
None	185 (37)		63 (45)		122 (34)		
Laboratory value							
Leucocyte count, 1000/µL	14 (9–20)	498	14 (10–17)	140	14 (9–22)	358	**0.010**
Procalcitonin, ng/ml	7 (1–26)	479	3 (1–12)	138	9 (2–32)	341	**< 0.001**
C- reactive protein, mg/l	189 (112–277)	405	197 (112–297)	105	182 (114–272)	300	0.471
Outcome parameters, n (%)							
28-day mortality, n (%)	136 (30)	456	25 (19)	130	111 (34)	326	**0.002**
Length of ICU/IMC stay, days	8 (4–18)	499	9 (4–17)	140	8 (4–18)	359	0.941
Need for mechanical ventilation	371 (74)		81 (57)		290 (81)		**< 0.001**
Length of mechanical ventilation, days	5 (2–12)	368	7 (2–14)	80	5 (2–12)	288	0.349
Length of anti-infective treatment, days	10 (5–17)	483	10 (6.5–17)	136	9 (5–17)	347	0.516
Need for RRT	133 (27)		27 (19)		106 (30)		**0.018**
Length of RRT, days	7 (3–15)	132	8 (3–21)	27	6 (3–15)	105	0.380

BMI: Body mass index; ICU: Intensive care unit; IMC: Intermediate care unit; RRT: Renal replacement therapy; SOFA: Sequential Organ Failure Assessment

Other infection sites included central nervous system infection, endocarditis

Data are reported as median (IQR) unless otherwise indicated and as n and %

Bold values are statistically significant for *p* ≤ 0.05

### sTREM-1 plasma concentrations and pre-existing conditions, organ dysfunction and outcome parameters

Non-survivors by day 28 presented with statistically significant higher sTREM-1 plasma concentrations at V1 (521 (CI;345–803) pg/mL vs. 327 (CI;215–529) pg/mL, *p* < 0.001) and V3 (438 (CI;333–630) pg/mL vs. 262 (CI;191–419) pg/mL, *p* < 0.001). This was also seen for patients with septic shock, the need for RRT, the need for mechanical ventilation or a positive SIC score at both days **(**Table 2, upper part of the table**)**. There was no statistical difference in sTREM-1 plasma concentrations at V1 or V3 whether it was a nosocomial nor an outpatient infection (*p* < 0.051 and *p* < 0.686, respectively). Whether the patient was immunosuppressed seemed to be relevant only at the beginning of disease at V1, so that there was no statistical difference at V3. Immunosuppressed patients presented with slightly but statistically significant higher sTREM-1 plasma concentrations at V1 (*p* < 0.045). If patients were included with a CCI score higher than the median score, sTREM-1 plasma concentrations were statistically significant higher at both timepoints (V1: 423 (CI;290–655) pg/mL vs. 320 (CI;213–535) pg/mL, *p* < 0.001) and V3: 354 (CI;230–571) pg/mL vs. 265 (CI;184–399) pg/mL, *p* < 0.001) **(**Table 2, lower part of the table, Suppl. Table 2). The delta in sTREM-1 plasma concentrations between V1 and V3 (ΔsTREM-1) did not statistically differ between survivors and non-survivors, patients with and without septic shock, RRT or mechanical ventilation. In addition, there was no statistical difference in ΔsTREM-1 whether the patient was male or not, if immunosuppression was present or absent or if the CCI score was higher than the median or not. Noteworthy, patients with an outpatient infection had a statistically significant higher ΔsTREM-1 than patients with a nosocomial infection (*p* < 0.001). sTREM-1 plasma concentrations decreased in median − 69.5 (IQR: −182;16.3) pg/mL in patients with an outpatient infection instead of in median − 22.7 (IQR:−105;41.5) pg/mL within a nosocomial infection (Suppl. Table 1). In comparison, there was no statistical difference in CRP decrease between V1 and V3 and only a trend in PCT decrease.

**Table 2:** sTREM-1 values in different outcomes vs. pre-existing conditions and comorbidities

Outcomes	sTREM-1 V1 pg/ml median (IQR)					*p* - value	sTREM-1 V3 pg/ml median (IQR)					*p* - value
	n	yes	*n*	no	*n*		*n*	yes	*n*	no	*n*	
Non-survivor by day 28	**456**	521 (345–803)	136	327 (215–529)	320	**< 0.001**	**398**	438 (333–630)	88	262 (191–419)	310	**< 0.001**
Septic Shock	**500**	406 (270–650)	359	293 (189–489)	141	**< 0.001**	**425**	346 (216–527)	299	236 (164–384)	126	**< 0.001**
Need for RRT	**500**	650 (434–862)	133	308 (207–475)	367	**< 0.001**	**425**	566 (394–766)	116	252 (176–371)	309	**< 0.001**
Need for mechanical ventilation	**500**	388 (257–650)	371	299 (220–469)	129	**< 0.001**	**425**	336 (217–531)	314	230 (168–396)	111	**< 0.001**
SIC Score: positive	**500**	547 (329–805)	234	347 (226–560)	266	**< 0.001**	**425**	396 (255–583)	196	292 (198–465)	229	**0.004**
Pre-existing conditions and comorbidities	**n**											
Male	**500**	362 (239–608)	337	371 (246–639)	163	0.604	**425**	302 (201–469)	288	303 (204–508)	137	0.684
Immunosuppression	**490**	403 (310–647)	45	357 (237–602)	445	**0.045**	**415**	402 (229–501)	36	296 (199–485)	379	0.159
Nosocomial	**500**	352 (226–588)	241	380 (249–639)	259	0.051	**425**	302 (209–495)	209	297 (192–494)	216	0.686
CCI: > median	**500**	423 (290–655)	234	320 (213–535)	266	**< 0.001**	**425**	354 (230–571)	196	265 (184–399)	229	**< 0.001**

CCI: Charlson Comorbidity Index; IQR: Interquartile range; RRT: Renal replacement therapy; SIC Score: Sepsis Induced Coagulopathy Score

Bold values are statistically significant for *p* ≤ 0.05

**Fig. 1:**
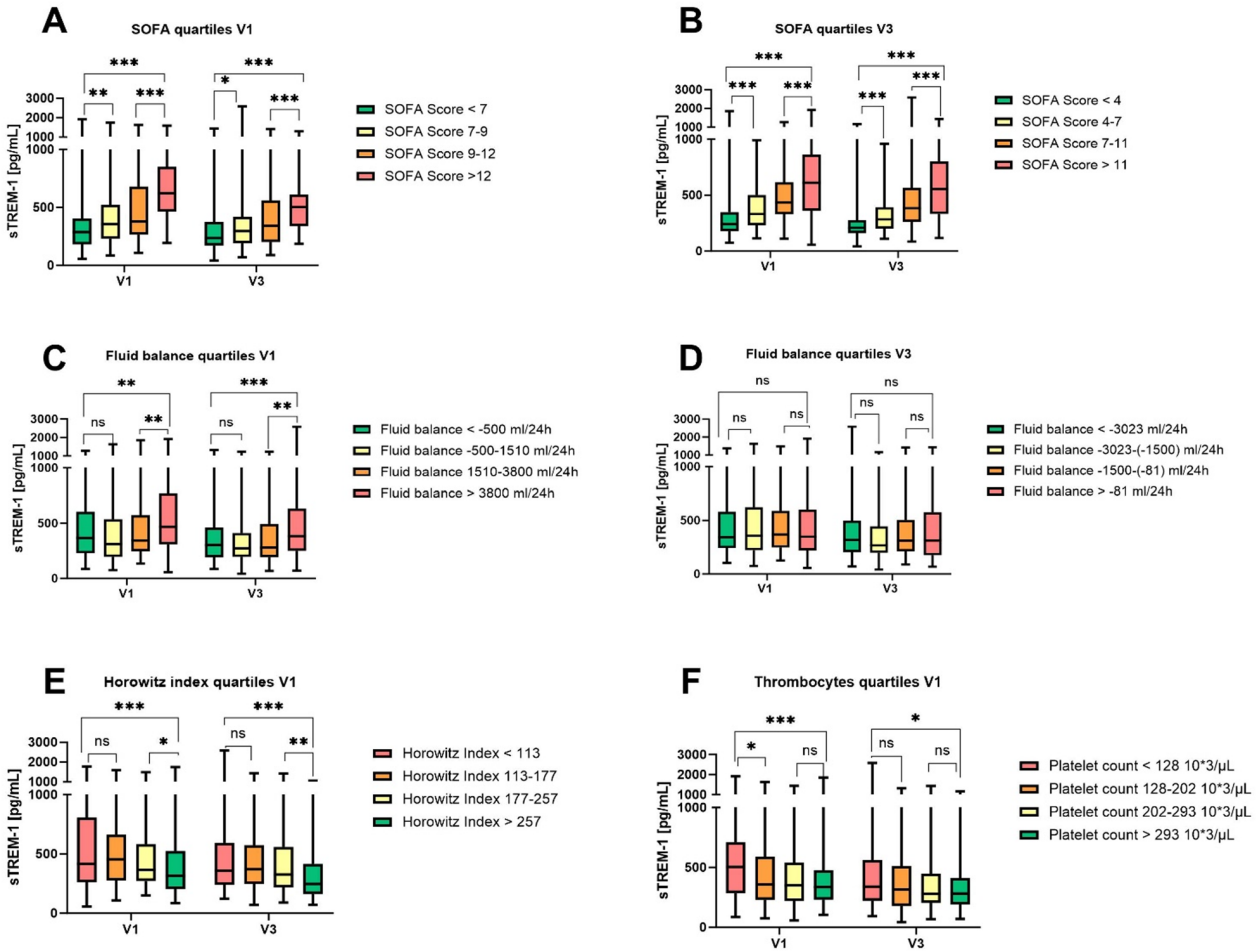
Organ dysfunction and sTREM-1 quartiles. Patients were grouped into quartiles according to their SOFA Score at V1 (**A**) and V3 (**B**), their fluid balance at V1 (**C**) and V3 (**D**), their Horowitz index at V1 (**E**) and their thrombocyte count at V1 (**F**). Horizontal line within the box marks the median, boxes depict the IQR, and whiskers indicate the total range. Group comparisons for individual comparisons were performed by two sided Mann–Whitney U-test. *with p-value ≤ 0.05; ** with p-value ≤ 0.01; *** with p-value ≤ 0.001. Abbreviations: sTREM-1: soluble Triggering receptor expressed on myeloid cells-1; SOFA Score: Sequential Organ Failure Assessment

**Fig. 2:**
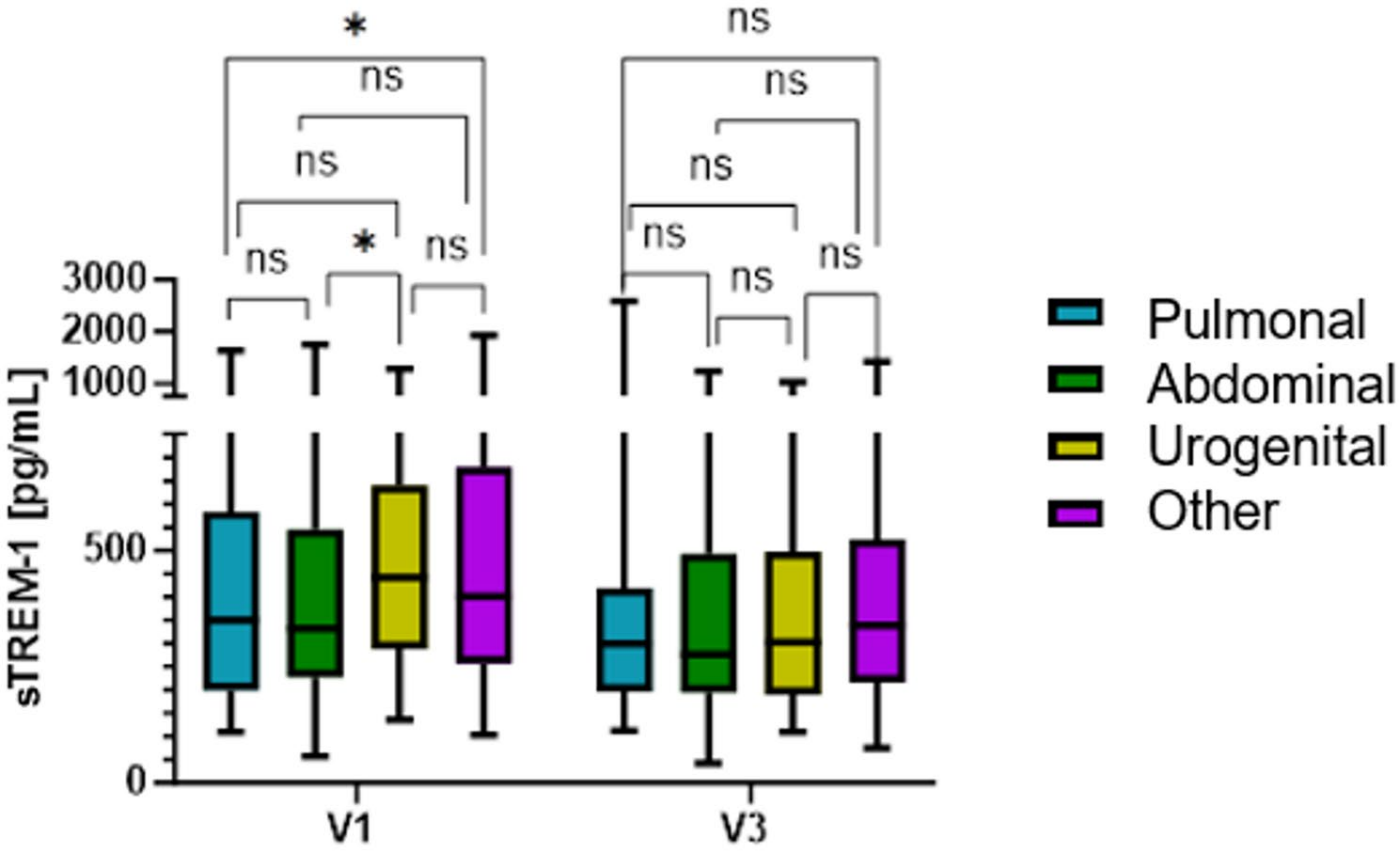
sTREM-1 values in different infection sites. Pulmonal focus presented as cyan bars (*n* = 118), abdominal focus as dark green bars (*n* = 153), urogenital focus as light green bars (*n* = 66) and other focus as purple bars (*n* = 163). Horizontal line within the box marks the median, boxes depict the IQR, and whiskers indicate the total range. Group comparisons for V1 and V3 were performed by Kruskal-Wallis test. Group comparisons for individual comparisons were performed by two sided Mann–Whitney U-test. * with p - value ≤ 0.05. Abbreviations: sTREM-1: soluble Triggering receptor expressed on myeloid cells-1

Patients were grouped according to their SOFA score, fluid balance, Horowitz index and thrombocyte count into quartiles. sTREM-1 plasma concentrations were highest (623 pg/mL (466–847)) in patients with SOFA scores above > 4th quartile. The difference in sTREM-1 plasma concentrations between all SOFA quartiles was statistically significant with *p* < 0.001 at both timepoints. For the SOFA score at V3 significant differences were also detected between the individual quartiles, with the differences at V3 being even more pronounced **(**Figs. 1A and 2B**)**. Concerning differences in sTREM-1 plasma concentrations in the fluid balance quartiles, there was only a statistically significant difference at V1. Patients with the highest positive fluid balance presented in median with very high sTREM-1 plasma concentrations. In the lower quartiles there was no statistical difference between the groups. At V3, sTREM-1 plasma concentrations were the same across all quartiles **(**Fig. 1C and D**).** Classification of patients according to Horowitz index quartiles at V1 revealed statistically significant different sTREM-1 plasma concentrations. Patients with a low Horowitz quotient (1st quartile) had statistically significant higher plasma concentrations than patients with a high Horowitz index (4th quartile) **(**Fig. 1E**).** Similar results were shown for the platelet count quartiles at V1. However, it should be emphasized that only the difference between the first quartile (low platelet count) and the 4th quartile (high platelet count) was statistically significant. Patients with high sTREM-1 plasma concentrations presented with a low platelet count **(**Fig. 1F).

### sTREM-1 plasma concentrations in different infection sites and among infections with different pathogens

Patients were grouped according to their main site of infection in the following groups: pulmonal (23.6%), abdominal (30.6%), urogenital (13.2%) and others (32.6%). sTREM-1 plasma concentrations differed marginally significant between groups at V1, whereas there was no statistical difference at V3. Patients with urogenital infections had statistically significantly higher sTREM-1 plasma concentrations than patients with abdominal infections at V1 (*p* = 0.027), however no statistical difference with further infection sites could be detected. In addition, patients with other infections had statistically significant higher sTREM-1 plasma concentrations than patients with pulmonal infections at V1 (*p* = 0.029). In all other cases, no statistical difference could be detected neither at V1 nor at V3 (Fig. 2).

**Fig. 3:**
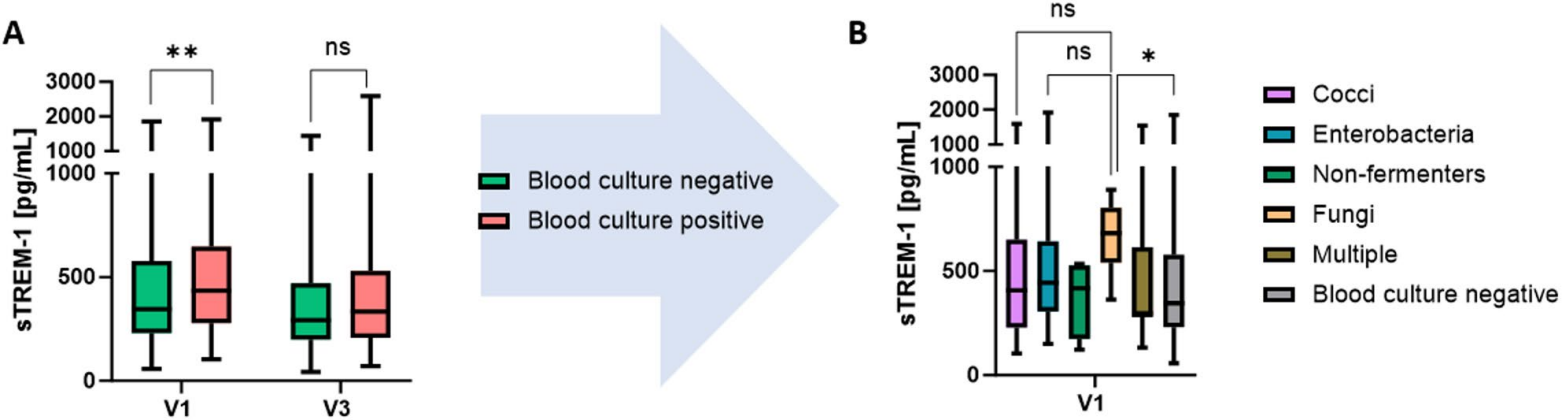
sTREM-1 values in infections with different pathogens. (**A**) Blood culture negative patients (*n* = 351, green bars) had statistically significant lower sTREM-1 plasma concentrations at V1 in comparison to patients with a bacterial or fungal growth in blood culture (*n* = 149, red bars). (**B**) sTREM-1 plasma concentrations among more specified different BC results at V1. Gram positive cocci are shown in light purple bars (*n* = 64), gram-negative *Enterobacteria* and Non-fermenters in cyan and dark green bars (*n* = 64, *n* = 4, respectively), fungi in orange bars (*n* = 6), multiple in brown bars (*n* = 11) and blood culture negative in grey bars (*n* = 351). Horizontal line within the box marks the median, boxes depict the IQR, and whiskers indicate the total range. Group comparisons for V1 and V3 were performed by Kruskal-Wallis test. Group comparisons for individual comparisons were performed by two sided Mann–Whitney U-test. *with p - value ≤ 0.05. Abbreviations: sTREM-1: soluble Triggering receptor expressed on myeloid cells-1

For analyses of blood culture results, patients were initially divided into two groups, those with and without a positive blood culture. Patients with a bacterial growth in the blood culture (*n* = 149 (29.8%) showed statistically significant higher sTREM-1 plasma concentrations at V1. This difference was no longer detectable at V3 **(**Fig. 3A).

There were no statistically significant differences in sTREM-1 values between patients with infections caused by different pathogens at V1 and V3. However, descriptive analyses revealed that patients with fungal bloodstream infections had the highest median sTREM-1 plasma concentrations at V1. In contrast, patients with infections caused by *Enterobacteria* exhibited the highest absolute sTREM-1 plasma concentrations (Fig. 3B).

### Outcome prediction analysis

Survival on day 28 could be predicted using sTREM-1 plasma concentrations at V1 with an AUC of 0.65 (95%CI 0.60–0.70, *p* < 0.001), outperforming CRP (AUC 0.55; 95%CI 0.49–0.61, *p* = 0.085) or PCT (AUC 0.53; 95%CI 0.48–0.58, *p* = 0.279). Predictive capacity of SOFA Score V1 concerning survival day 28 was similar to sTREM-1 (AUC 0.64; 95%CI 0.59–0.69, *p* < 0.001) in our cohort **(Suppl. Figure 1)**. For predicting need for RRT the best predictive performance was achieved using sTREM-1 plasma concentrations V1 with an AUC of 0.81 (95%CI 0.77–0.88, *p* < 0.001), outperforming the established functional marker creatinine (AUC 0.73; 95%CI 0.68–0.78, *p* < 0.001) and the newly described kidney damage marker soluble form of urokinase-type plasminogen activator receptor (suPAR) (AUC 0.68; 95%CI 0.63–0.73, *p* < 0.001) (Suppl. Figure 2).

### Cut-offs

In the phase IIb study by Francois et al. [9] to test the TREM-1 inhibitor nangibotide (Inotrem, Paris, France), the cut-off of 400 pg/mL was used to differentiate between a group of patients with high and low sTREM-1 plasma concentrations. However, only patients with a sTREM-1 level of over 532 pg/mL benefited with a significant SOFA score reduction (outcome parameter of the study). Therefore, we tested both cut-offs in this study to examine whether there were statistically significant differences between the outcomes, disease severity parameters or pre-existing conditions **(**Table 3**).** Regardless of whether the cut-off of 400 pg/mL or the higher cut-off of 532 pg/mL was used, patients with sTREM-1 plasma concentrations above both cut-offs revealed a statistically significant higher 28-day mortality (*p* < 0.001), incidence of septic shock (*p* < 0.001), need for renal replacement therapy (*p* < 0.001) or mechanical ventilation (*p* < 0.001). Concerning the type of infection, patients showed a trend towards a higher rate of nosocomial infections when sTREM-1 plasma concentrations at V1 were above 532 pg/mL (*p* = 0.033), whereas there was no statistical difference when the cut-off of 400 pg/mL was used (*p* = 0.154). For disease severity parameters, there were statistically significant differences for the SOFA score at V1 und V3 regardless of which cut-off was used (*p* < 0.001). Patients with sTREM-1 plasma concentrations above one of the two cut-offs showed a high comorbidity burden in both cases (*p* < 0.001, *p* = 0.004 respectively).

**Table 3:** Outcomes, disease severity and pre-existing conditions depending on different cut-off values

		sTREM-1 > 400 pg/mL	sTREM-1 ≤ 400 pg/mL	*p* -value	sTREM-1 > 532 pg/mL	sTREM-1 ≤ 532 pg/mL	*p* – value
Non-survivor by day 28	yes	43.6	18	**< 0.001**	45.6	22.3	**< 0.001**
	no	56.4	82		54.4	77.7	
Septic Shock	yes	80.1	65	**< 0.001**	81.2	67.4	**0.001**
	no	19.9	35		18.8	32.6	
Need for RRT	yes	53.1	9.9	**< 0.001**	54.4	13.5	**< 0.001**
	no	46.9	90.1		45.6	86.5	
Need for mechanical ventilation	yes	81.4	68.2	**< 0.001**	83.8	69.7	**< 0.001**
	no	18.6	31.8		16.2	30.3	
Nosocomial	yes	44.7	51.1	0.154	41.2	51.5	0.033
	no	55.3	48.9		58.8	48.5	
Immunosuppression	yes	10.4	8.2	0.395	10.9	8.4	0.369
	no	89.6	91.8		89.1	91.6	
SOFA score V1:	Median (IQR)	11 (8–13)	8 (6–10)	**< 0.001**	11 (9–13)	8 (6–10)	**< 0.001**
SOFA score V3:	Median (IQR)	10 (7–13)	6 (3–8)	**< 0.001**	10 (7–14)	6 (3–9)	**< 0.001**
CCI:	Median (IQR)	3 (2–5)	2 (1–4)	**0.001**	3 (2–5)	2 (1–4)	**0.004**

*Abbreviations:* CCI: Charlson Comorbidity Index; SOFA Score: Sequential Organ Failure Assessment

sTREM-1: soluble Triggering receptor expressed on myeloid cells-1, RRT: renal replacement therapy present and absent in [%]

Bold values are statistically significant for *p* ≤ 0.05

### Survival in patients with high sTREM-1 plasma concentrations

**Fig. 4:**
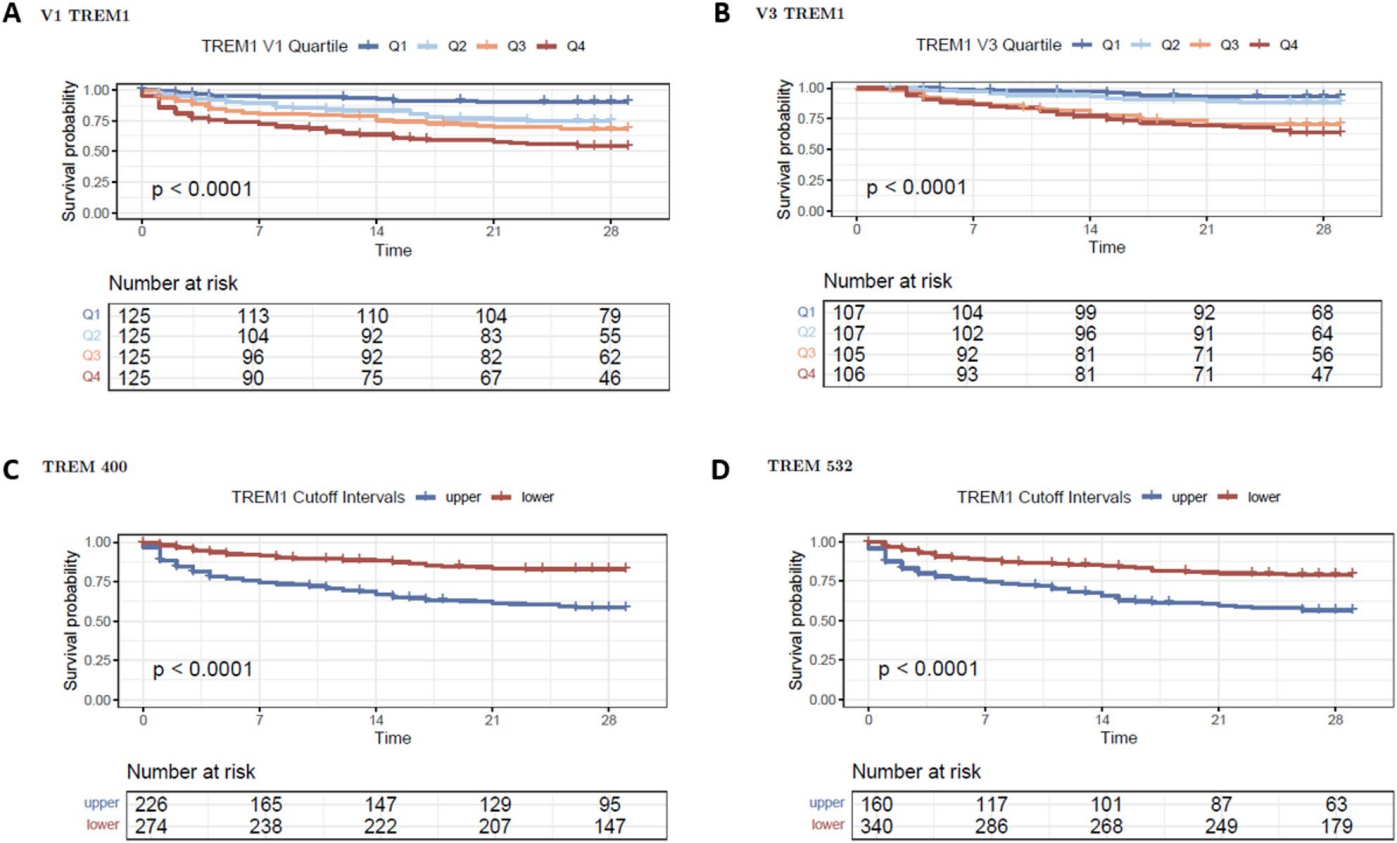
Kaplan Meier Curves of sTREM quartiles and cut-offs. **A** and **B.**) Patients grouped according to their sTREM-1 plasma concentration quartiles at V1 and V3, respectively (Q1 presented in dark blue lines, Q2 in light blue, Q3 in orange and Q4 in red); **C** and **D.**) patients are grouped according to the sTREM-1 cut-offs of > 400 and > 532 pg/mL respectively; Survival probability is presented in all subfigures until day 28; the survival probability differs statistically significant in all analysis with *p* < 0.0001. Abbreviations: sTREM-1: soluble Triggering receptor expressed on myeloid cells-1

Patients with sTREM-1 plasma concentrations at V1 in the 4th quartile only survived in up to 50% at day 28. In contrast, patients with values within the first quartile survived in almost 100% until day 28 (Fig. 4A). In case of sTREM-1 plasma concentrations above the median at V3, almost 50% of these patients died by day 28 (Fig. 4B). This was statistically significant for both timepoints.

Using the two sTREM-1 cut-off values of 400 pg/mL and 532 pg/mL, it was demonstrated that there is no statistically significant difference in survival between patients with sTREM-1 levels above 400 pg/mL and those above 532 pg/mL. However, sTREM-1 levels exceeding 400 pg/mL are associated with a statistically significant higher probability of non-survival by day 28 compared to levels below 400 pg/mL (Fig. 4C and D).

The best cut-off plasma concentration of sTREM-1 to differentiate between survival and non-survival on day 28 was 408 (CI; 297;505) pg/ml in our study.

## Discussion

### sTREM-1: a disease severity marker that fits for all?

In our cohort, sTREM-1 plasma concentrations were significantly higher in patients with septic shock or various organ failures and in non-surviving patients. This study therefore showed that sTREM-1 meets the typical criteria for a disease severity parameter, especially since there was no correlation with established inflammatory markers such as CRP and PCT (Suppl.). Furthermore, the sTREM-1 release seems to be partly dependent on the underlying infection site, since sTREM-1 plasma concentrations were significantly higher in patients with urogenital infections than in patients with other infection sites at V1. However, these differences were no longer detectable at V3. Similarly, the data on the underlying pathogens further support the hypothesis that the type of infection might not play a decisive role in TREM-1 activation, even though infections with certain gram-negative pathogens led to higher sTREM-1 levels. This is in contrast to a study on Host-Derived Delta-Like Canonical Notch Ligand-1 (DLL-1), a receptor that also occurs on monocytes and vascular endothelium, which revealed a clearer difference concerning infection sites [23]. Patients with high DLL-1 levels showed significantly more frequent infections with certain pathogens such as *S. aureus*, *E. coli* or fungi and a clear difference in DLL-1 levels at the different sites of infection [23]. Similar results, showing that other new sepsis biomarkers are more likely to show an organ system or infection site specific increase in plasma concentrations, were obtained from a study on receptor for advanced glycation end products (RAGE). RAGE is specifically elevated in patients with acute respiratory distress syndrome (ARDS) or pulmonary sepsis [24, 25]. Interestingly, our study showed that patients suffering from an outpatient infection had a significantly higher decrease in sTREM-1 plasma concentrations from day 1 to 3 than in patients with a nosocomial infection, possibly due to a higher likelihood of surgical source control. In 2005, Gibot et al. showed that patients with a high sTREM-1 plasma concentration at disease onset had a higher survival rate [26]. This is in contrast to our data, where patients with high sTREM-1 plasma concentrations at V1 and V3 had a significantly higher mortality rate by day 28. However, it is possible that the decrease in sTREM-1 plasma concentrations is relevant here and that patients with a rapid sTREM-1 clearance are more likely to survive. In our study, however, a high sTREM-1 plasma concentration at disease onset was not a protective factor. The role of sTREM-1 kinetic as a biomarker of response needs to be investigated in further studies, especially with regard to future targeted therapies.

Another aspect that makes sTREM-1 a suitable disease severity parameter for a general sepsis population is that there was a significant increase of sTREM-1 plasma concentration in patients with various organ failures in this study (Table 2; Fig. 1). This was shown by statistically significant higher sTREM-1 plasma concentrations in patients with the need for RRT, invasive ventilation or a positive SIC score. Furthermore, patients with a low Horowitz Index and a PLC SIC subscore showed sTREM-1 plasma concentrations in the highest quartile of the study population. At V1, patients with the highest positive fluid balance also showed the highest sTREM-1 plasma concentrations, indicating TREM-1 activation in terms of hemodynamic dysfunction. Regarding the general organ dysfunction SOFA score, it was impressively shown that the higher the SOFA score, the higher the sTREM-1 plasma concentrations of the patients. The sTREM-1 elevation therefore seems to be related to the degree of organ damage, (e.g. the lungs, kidneys, circulation and coagulation), whereas pre-existing conditions such as sex or immunosuppression did not play a role. It was only shown that patients with a CCI higher than the median CCI had increased plasma concentrations of sTREM-1. However, the burden of comorbidities was low as the median CCI in the overall cohort was only 2 (IQR 1–4). In addition, it should also be mentioned that TREM-1 has been implicated in many sterile inflammatory processes, like chronic liver injury and fibrosis [18], cardiovascular [19] and cerebrovascular diseases [20]. All together, these observations fit with the finding that sTREM-1 seems to be a sterile organ damage marker and its elevation is not limited to infectious disease [27]. This needs to be considered in future when using sTREM-1 as a sepsis biomarker.

However, given that sTREM-1 is elevated in various forms of organ failure and seems to be not specific to a single organ system damage, it is plausible that sTREM-1 could contribute to the stratification of different subgroups within sepsis. Since high sTREM-1 levels are associated with strong activation of the innate immune system, the inseparably linked coagulation system is also (systemically) co-activated [28, 29], inducing SIC. In line with that, SIC has been shown to be associated with increased mortality and morbidity [22, 30]. Our finding, that increased sTREM-1 plasma concentrations correlate with increased SIC scores, might qualify sTREM-1 as a potential biomarker for sepsis phenotypes characterized by impaired coagulation. Thus, it could help optimize participant selection in clinical intervention studies treating sepsis-induced coagulopathy. The need for such novel, optimized recruitment strategies for trials treating sepsis-induced coagulopathy has recently been called for by several leading scientific groups [28, 29]. Furthermore, studies on sTREM-1 concentrations in urine showed that urinary sTREM-1 measurement can be a useful tool in diagnosing sepsis and provide an early warning of possible secondary sepsis-associated acute kidney injury (AKI) [31, 32]. However, it is not yet clear whether the increase in urine in patients with sepsis or sepsis-associated AKI is due to increased excretion reflecting elevated plasma concentrations, an impaired clearance or whether it is a direct sTREM-1 formation in the urogenital system locally produced by the endothelial cells or the infiltrating inflammatory cells that are recruited during acute tubular necrosis or both [33]. Furthermore, a release from the kidneys into the bloodstream is discussed in the literature [27]. A study of plasma and urine concentrations of sTREM-1 in patients with renal and non-renal systemic lupus erythematosus (SLE) showed that patients with an acute renal involvement had higher sTREM-1 plasma concentrations in urine but not in plasma. On the other hand, patients without an AKI showed significantly higher plasma concentrations, which led to a higher urine concentration than in healthy controls, however, did not reach urine concentrations of sTREM-1 like in patients with renal SLE [34]. In our study, there was a moderate correlation between sTREM-1 at V1 and creatinine at V1 (Suppl.; Correlation analyses), which emphasizes the importance of including pre-existing organ dysfunction more strongly in the evaluation of sTREM-1 in sepsis in the future. In addition, sTREM-1 showed best predictive performance for need of RRT outperforming routinely used and newly described biomarkers in our cohort, indicating a more detailed investigation of the relationship between kidney function and sTREM-1 is urgently needed.

### Cut offs

In the phase 2b study by Francois et al., the same ELISA was used to measure sTREM-1 plasma concentrations as in our study. They applied a sTREM-1 cut-off of 400 pg/mL, as this was determined to be an appropriate cut-off in preliminary studies. Since this study showed that only patients with a sTREM-1 level of greater than 532 pg/mL reached the primary endpoint (change in SOFA score) [9], we tested both cut-off values concerning their potential for the prediction of outcome parameters and preliminary conditions. Even with a cut-off of over 400 pg/mL, patients had a significantly higher probability of suffering from septic shock, reaching higher SOFA score values or non-survival. Furthermore, these patients also had a higher burden of comorbidities indicating higher plasma concentrations of sTREM-1 before the onset of sepsis due to their non-infectious preexisting disease with a certain organ damage. There was no difference when using the higher cut-off of 532 pg/mL. Therefore, the 400 pg/mL cut-off seems to be suitable for the differentiation in high and low sTREM-1 plasma concentrations for treatment guidance in future studies. Furthermore, the change in the SOFA score is a soft outcome parameter: Some authors have described the clinical importance of this change as uncertain [35], therefore, these results regarding the use of a higher cut-off should not be overemphasized. In addition, in our study we also confirmed 408 pg/mL as the best cut-off (Youden criteria) for distinguishing between survival and non-survival by day 28. However, in a cohort of febrile African children at risk of sepsis, three cut-off groups were described for risk stratification with regard to non-survival. Children with sTREM-1 levels above 629 pg/mL (high-risk group) showed a mortality rate of 31.9%, whereas children with sTREM-1 levels between 239 and 629 pg/mL (intermediate risk group) died only in 3,2% of cases [36]. In a recently published study on postoperative patients following abdominal surgery, a cut-off value of 346 pg/mL was established for risk assessment of septic shock [37]. Accordingly, cut-off values for effective risk stratification and treatment guidance need to be investigated more thoroughly for specific patient groups to select suitable patients for targeted therapy.

The exploratory nature of our study is a limitation that needs to be addressed. Furthermore, data were exclusively collected in the ICU setting, limiting the analysis to critically ill patients. As a result, no information is available on septic patients with less severe disease who could be managed in general wards. Another limitation of the study is, that we did not assess pre-existing comorbidities and associated organ dysfunctions individually; instead, we used the CCI score for this purpose. Furthermore, there is no data on the commonly used scores SAPS II or APACHE II available. Consequently, the role of sTREM-1 as a marker of organ damage and disease severity, as well as the impact of pre-existing comorbidities on patient selection for targeted therapy, needs further investigation.

## Conclusion

These results led us to conclude that the elevation of sTREM-1 in septic patients particularly occurs in those with a high degree of organ damage, who are at high risk for adverse outcomes. In addition, the primary site of infection may play a role in its elevation. It is, therefore, suitable as a biomarker in patients with different causes of sepsis and thus a promising therapeutic target. A cut-off of greater than 400 pg/mL has proven to be a good threshold value in various studies and might be used in future studies.

## Supplementary Information

Supplementary material 1

## Data Availability

Upon reasonable request available.

## Abbreviations

AKI: Acute kidney injury
APACHE II: Acute Physiology And Chronic Health Evaluation-II
ARDS: Acute respiratory distress syndrome
BC: Blood culture
CCI: Charlson Comorbidity index
CRP: C-reactive protein
DLL-1: Host-Derived Delta-Like Canonical Notch Ligand-1
ELISA: Enzyme-linked immunosorbent assay
ICU: Intensive care unit
IMC: Intermediate care unit
IQR: Interquartile range
LPS: lipopolysaccharide
MAMPs/DAMPs: Microbial/damage-associated molecular patterns
PCT: Procalcitonin
PLC: Platelet count
PRRs: Pattern recognition receptors
PSSC: Sum of the platelet count SIC subscore
RAGE: Receptor for advanced glycation end products
RRT: Renal replacement therapy
SAPS: Simplified Acute Physiology Score
SIC: Sepsis-induced coagulopathy
SIRS: Systemic inflammatory response syndrome
SLE: Systemic lupus erythematosus
sTREM-1: Soluble triggering receptor expressed on myeloid cells-1
suPAR: Soluble form of urokinase-type plasminogen activator receptor
TREM-1: Triggering receptor expressed on myeloid cells-1
ΔsTREM-1: Difference of sTREM-1 between V1 and V3
