# Supplementary material for: Soluble triggering receptor expressed on myeloid cells-1 as a predictive biomarker in sepsis: insights into cut-offs, mortality risk, and treatment guidance—secondary analysis of the Next GeneSiS-Trial
Source: Crit Care. 2026 Apr 21;30:219. doi: 10.1186/s13054-026-06025-6 (PMC13126823; doi:10.1186/s13054-026-06025-6)
Supplement: Supplementary file 1 — Supplementary material 1 [file 13054_2026_6025_MOESM1_ESM.docx]

**Supplements**

***Correlation analyses****:* Plasma concentrations of sTREM-1 at V1 and V3 correlated positively (r=0.63). There was no correlation with inflammation markers like PCT and CRP at V1 (with PCT V1 r=0,23 and with CRP V1 r=0,09) and at V3 (with PCT V3 r=0,23; with CRP V3 r=0.16). Regarding organ dysfunction parameters, plasma concentrations of sTREM-1 V1 correlated weakly with the SOFA score at V1 (r=0.35) and sTREM-1 V3 correlated weakly with the SOFA score at V3 (r=0.48). Plasma concentrations of sTREM-1 at V1 correlated moderately with creatinine V1 (r=0.57) and sTREM-1 V3 correlated only slightly with creatinine at V3 (r=0.32). There was no correlation of sTREM-1 plasma concentrations with the fluid balance at V1 or V3 nor with the Horowitz index at V1 or V3. Plasma concentrations of sTREM-1 at V1 correlated weakly with thrombocytes (r=0.45), whereas there was no correlation at V3.

***Suppl. Table 1.) ΔsTREM-1 values in different outcomes vs. pre-existing conditions***

|  |  | ΔsTREM-1 pg/ml  median (IQR) | | | | p - value |
| --- | --- | --- | --- | --- | --- | --- |
| Outcomes | **n** | yes | n | no | n |  |
| 28-day mortality | **398** | -24.2 (-175-64.6) | 88 | -43.4 (-127-25.4) | 310 | 0.369 |
| Septic Shock | **425** | -44.6 (-159-37.4) | 299 | -50.3 (-124-10.5) | 126 | 0.897 |
| Need for RRT | **425** | -82.5 (-229-105) | 116 | -42.2 (-122-15.4) | 309 | 0.732 |
| Need for mechanical ventilation | **425** | -38.4 (-153-40.1) | 314 | -63.9 (-139-2.68) | 111 | 0.065 |
| SIC Score: positive | **409** | -80.7 (-250-25-4) | 65 | -43.2 (-123-31.7) | 344 | 0.139 |
|  |  |  |  |  |  |  |
| Pre-existing conditions | **n** |  |  |  |  |  |
| Male | **425** | -39.6 (-126-30.1) | 288 | -60.2 (-174-31.7) | 137 | 0.359 |
| CCI: > median | **499** | -55.1 (-175-32) | 196 | -40.1 (-122-27.4) | 229 | 0.379 |
| Immunosuppression | **415** | -62.5 (-153-19.1) | 36 | -44.4 (-148-31.4) | 379 | 0.339 |
| Nosocomial | **425** | -22.7 (-105-41.5) | 209 | -69.5 (-182-16.3) | 216 | **<0.001** |

Abbreviations: CCI: Charlson Comorbidity Index; IQR: Interquartile range; RRT: renal replacement therapy; SIC Score: Sepsis Induced Coagulopathy Score; ΔsTREM-1: difference of sTREM-1 plasma concentrations from day 1 to day 3
Bold values are statistically significant for p ≤ 0.05

***Suppl. Table 2.) sTREM-1 values in different outcomes vs. pre-existing conditions and comorbidities in sepsis vs. septic shock***

|  |  | Sepsis  sTREM-1 V1 pg/ml  median (IQR) | | | | p - value |  | Septic shock  sTREM-1 V1 pg/ml  median (IQR) | | | | p - value |
| --- | --- | --- | --- | --- | --- | --- | --- | --- | --- | --- | --- | --- |
| Outcomes | **n** | yes | n | no | n |  | **n** | yes | n | no | n |  |
| 28-day mortality | **141** | 359 (246-540) | 36 | 264 (179-405) | 105 | 0.033 | **359** | 516 (329-761) | 144 | 346 (242-576) | 215 | **<0.001** |
| Need for RRT | **141** | 538 (407-816) | 27 | 241 (170-381) | 114 | **<0.001** | **359** | 674 (451-863) | 106 | 335 (239-502) | 253 | **<0.001** |
| Need for MV | **141** | 305(194-493) | 81 | 281 (171-469) | 60 | 0.662 | **359** | 436 (283-680) | 290 | 316 (237-469) | 69 | **<0.001** |
| SIC Score: positive | **135** | 600 (246-883) | 14 | 273 (184-407) | 121 | **0.007** | **347** | 544 (329-797) | 70 | 369 (254-617) | 277 | **0.003** |
|  |  |  |  |  |  |  |  |  |  |  |  |  |
| Pre-existing conditions and comorbidities |  |  |  |  |  |  |  |  |  |  |  |  |
| Male | **141** | 305 (193-477) | 93 | 263 (176-496) | 48 | 0.797 | **359** | 391 (267-640) | 244 | 414 (287-654) | 115 | 0.379 |
| Immunosuppression | **141** | 517(331-1248) | 8 | 274 (187-469) | 133 | 0.020 | **349** | 403 (310-642) | 37 | 407 (269-650) | 312 | 0.553 |
| Nosocomial | **141** | 278 (191-407) | 77 | 303 (188-533) | 64 | 0.484 | **359** | 371 (257-630) | 164 | 421 (274-670) | 195 | 0.152 |
| CCI: > median | **141** | 353 (220-538) | 65 | 246 (162-388) | 76 | **0.007** | **359** | 478 (310-691) | 169 | 364 (240-582) | 190 | **<0.001** |

Abbreviations: CCI: Charlson Comorbidity Index; IQR: Interquartile range; MV: mechanical ventilation, RRT: renal replacement therapy; SIC Score: Sepsis Induced Coagulopathy Score
Bold values are statistically significant for p ≤ 0.05


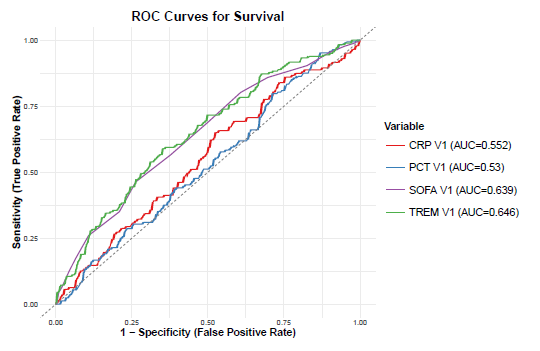


**Suppl. Figure 1:** AUC curves of sTREM-1, SOFA Score, CRP and PCT V1 in predicting survival day 28

Abbreviations: CRP: C-reactive protein, PCT: procalcitonin, SOFA: Sequential Organ Failure Assessment, TREM-1: Triggering receptor expressed on Myeloid Cells-1: ROC: Receiver Operating Characteristics


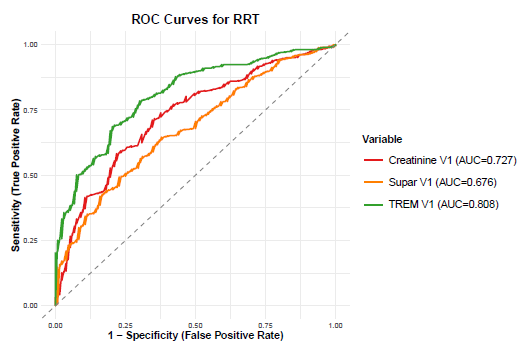


**Suppl. Figure 2:** AUC curves of sTREM-1, suPAR and Creatinine V1 in predicting RRT

Abbreviations: ROC: Receiver Operating Characteristics; RRT: renal replacement therapy; suPAR: Soluble form of urokinase-type plasminogen activator receptor TREM-1: Triggering receptor expressed on Myeloid Cells-1:
